# Narrative Potential of Picture-Book Apps: A Media- and Interaction-Oriented Study

**DOI:** 10.3389/fpsyg.2020.593482

**Published:** 2020-12-02

**Authors:** Claudia Müller-Brauers, Christiane Miosga, Silke Fischer, Alina Maus, Ines Potthast

**Affiliations:** ^1^Department Didaktik der Symbolsysteme - Schwerpunkt Deutsch (Didactics of Symbol Systems - German), Institute for Special Education, Leibniz University Hannover, Hanover, Germany; ^2^Department Sprach-Pädagogik und -Therapie (Department of Speech and Language Pedagogy and Therapy), Institute for Special Education, Leibniz University Hannover, Hanover, Germany

**Keywords:** picture-book apps, app analysis, digital shared reading, interaction analysis, early story comprehension

## Abstract

Digital literature is playing an increasingly important role in children's everyday lives and opening up new paths for family literacy and early childhood education. However, despite positive effects of electronic books and picture-book apps on vocabulary learning, early writing, or phonological awareness, research findings on early narrative skills are ambiguous. Particularly, there still is a research gap regarding how app materiality affects children's story understanding. Thus, based on the ViSAR model for picture-book app analysis and data stemming from 12 digital reading dyads containing German monolingual 2- to 3-year-olds and their caregivers this study assessed the narrative potential of a commercial picture-book app and how this is used in interaction. Results of the media analysis showed that the app provides a high number of narrative animations. These animations could be used interactively to engage the child in the story. However, results of the interaction analysis showed that adult readers do not exploit this potential due to their strong concentration on operative prompts and instructions. Furthermore, an explorative analysis of the relation between adults' utterances and children's story comprehension provided preliminary indicators regarding how the length of reading duration and the number of utterances might relate to children's understanding of the story. Findings and methodological limitations of the study are discussed and combined didactically with practical recommendations on how to use narrative animations in interaction effectively.

## Introduction

Due to increasing media use across societies, even very young children's media experiences have changed fundamentally (Ofcom, 2018, 2019; Bitkom, 2019). From an early age, children begin to explore digital media and use them very intuitively (Neumann, 2014; Reid-Chassiakos et al., 2016; Neumann and Neumann, 2017). As a consequence, digital technologies are expanding children's early access to written language by establishing modified modes of communication in home literacy environments (McPake et al., 2013; Aliagas and Margallo, 2015). The international market is reacting to this trend with a wide range of apps targeted at children (Sari et al., 2019; Starke et al., 2020). Besides gaming and entertainment apps, there is an increasing number of educational apps, storybook apps, and electronic books addressing children's language and literacy development in preschool age (Sargeant, 2015; Sari et al., 2019). Due to their flexible usability, technical features, and easy applicability (Serafini et al., 2016), these have become increasingly popular with parents and are supplementing print-based literacy activities in children's everyday lives (Ehmig and Reuter, 2013; Neumann, 2014; Ólafsson et al., 2014; Kabali et al., 2015; Real and Correro, 2015; Kucirkova and Littleton, 2016; Chaudron et al., 2018). In contrast to print-based picture books, picture-book apps contain visual and audio animations (visual movements, images, or sounds) that can be controlled by the touchpad either with or without the help of a visual pointer—a so-called hotspot (Serafini et al., 2016). They can also include technical features such as gaming activities, navigation applications, videoclips, or recording functions (Sargeant, 2015; Aguilera et al., 2016; Serafini et al., 2016).

### Digital Stories and Early Literacy

Numerous international studies have shown that emergent literacy skills unfold in reciprocity with children's literature (Whitehurst et al., 1988; Sénéchal and LeFevre, 2001; Dickinson et al., 2012; Towson et al., 2017) because even very young children not only develop a natural interest in all various forms of picture books (Kümmerling-Meibauer and Meibauer, 2005), but also expand their linguistic knowledge and early literacy skills in dialogic reading situations (Justice and Ezell, 2000; Blewitt et al., 2009; Grolig et al., 2019; Clemens and Kegel, 2020). Children benefit from the multimodality^1^ of parental reading styles. In dialogic reading (Whitehurst et al., 1988), adults not only act intuitively to (re-)establish attention or adapt their non-verbal and verbal behavior to the needs and interests of the child (Hargrave and Sénéchal, 2000). They also ensure comprehension by applying multimodal strategies of “communicative attunement” (Stern, 2000, pp. 138–139) and “sustained shared thinking” (Siraj-Blatchford, 2007, p. 17, 18) including questioning and prompts that highlight relevant verbal information (Hildebrandt et al., 2016) and help children to extract and process information on what is told and how the story evolves (Strouse et al., 2013; Hoffman and Paciga, 2014). When reading storybooks, adult readers also introduce children to motifs, figures, narrative themes (Kümmerling-Meibauer and Meibauer, 2015), and story schemes that they reproduce in their own retellings based on mental representations (Fox, 1993). Especially decontextualized talk significantly facilitates children's language skills (Rowe, 2012; Demir et al., 2015). Decontextualized talk comprises talk beyond the “here and now” of the immediate context such as explanation, pretending, or narrative talk (Rowe, 2012) about the past and future, absent objects, or abstract entities, or explanations of cause-and-effect relations (Curenton et al., 2008; Demir et al., 2015). Different approaches define this concept across a range of dimensions, so that different studies operationalize it in various ways (see Grimminger et al., 2019, for an overview). Moreover, reading digital stories to children also holds numerous potentials for the development of early literacy. Ihmeideh (2014) assessed the efficacy of electronic book reading during preschool age on early literacy domains such as print awareness, vocabulary, phonological awareness, and alphabetic knowledge. Results showed higher early literacy scores in the experimental group after electronic book intervention than in the control group that used traditional book material. Several studies have also reported positive effects of digital storybooks on early literacy skills and language development (Shamir and Korat, 2007; Shamir and Shlafer, 2011; Shamir and Baruch, 2012; Neumann, 2014; Neumann and Neumann, 2017; Strouse and Ganea, 2017; Zipke, 2017; Herodotou, 2018; Lee, 2020). However, there is still a lack of research on how the specific conditions in digital reading impact children's understanding of stories and how shared reading interaction and adults' responsive strategies during digital reading are affected by the digital device (Herodotou, 2018; Courage, 2019). In an intervention study, Shamir and Korat (2007) compared the impact of digital story reading in comparison to analog storybook reading on the early literacy skills of 128 children aged 5–6 years from low- and middle-SES backgrounds. Post-measurements showed no differences in children's story comprehension performances in the two conditions analog vs. digital. Smeets and Bus (2015) assessed the effects of different types of autonomously operated electronic books on vocabulary learning and story comprehension in preschool children aged 4–5 years: (a) static electronic books with an activated reading-aloud function and no visual or audio animations, (b) animated electronic books with a reading-aloud function and visual and audio animations, and (c) interactively animated electronic books with a reading-aloud function and integrated hotspots presenting unknown words after being activated. Whereas children's vocabulary benefited most from interactively animated electronic book reading, there were no clear positive effects on early story comprehension. Moreover, children's story comprehension did not differ between the conditions. Zhou and Yadav (2017) also reported no significant effect of multimedia or digital story reading on story comprehension in preschool age. In contrast, Korat (2009, 2010) found positive effects of digital storybook reading on story comprehension in preschool age based on a non-commercial electronic book story specially designed to optimize literacy learning for study purposes. In a study with 3- and 5-year-old children, Parish-Morris et al. (2013) reported a negative impact of app-related technical features on children's early story comprehension and parental language teaching strategies in shared reading situations. In other words, in the digital condition, Parish-Morris et al. (2013) quantified a lesser degree of dialogic reading strategies based on content-related utterances and prompts and a higher degree of instructive utterances addressing the child's behavior. Vice versa, in the analog reading situation and in a situation in which the technical features of the app were deactivated, they found a lesser degree of behavior-related talk and a higher amount of content-related talk. Krcmar and Cingel (2014) also found that when reading analog story books to their 2- to 5-year-olds, parents focused more on content aspects, got less distracted by technical features, and showed more responsiveness and engagement by adjusting to children's questions or comments on the story compared to the digital condition. In a study with children aged 2–3 years, Miosga (2020) showed that under digital reading conditions, the presence of technical features has a negative influence on not only language teaching strategies but also children's abilities to understand the story. In comparison to the analog condition, in the digital condition, adults used more media-related and less content-related utterances and proved to be less emotionally attuning and cognitively activating when interacting with their children. Negative effects on story comprehension were also found when storybooks contained numerous gaming items and hotspots (Yokota and Teale, 2014). In contrast, a meta-analysis based on 29 experimental studies by Takacs et al. (2014) revealed that when listening to multimedia stories, young children benefit to a higher degree in terms of story comprehension compared to traditional print-based story reading settings that are not framed interactively by an adult, provided that electronic storybooks are not overloaded with technical features. Other studies also report a positive influence of digital reading on story and reading comprehension, but these studies targeted older children and used outdated technologies (e.g., Doty et al., 2001).

### The Role of the Medium

In sum, empirical evidence shows that digital reading enhances literacy learning in various ways. With respect to story comprehension, research findings are heterogeneous. Studies indicate that early story comprehension can be affected negatively by app materiality and the specific interactive conditions of digital reading, but reading digital stories can also foster children's narrative and linguistic skills (Verhallen et al., 2006). Note that, interactivity in digital reading situations is not identical to that under analog book reading conditions. Thus, adult readers not only have to guide the reading situation dialogically, they also have to operate the technical features of the app in interplay with the narrative (Müller-Brauers et al., 2020). “Readers of digital picture books must work through the presentation of a fictional narrative using physical, cognitive, visual, emotional, and embodied strategies and capabilities, among others” (Serafini et al., 2016, p. 510). In this respect, the question arises, however, whether the technical features of an app (hotspots, video clips, background music) and the way animations are linked to the story also play a role in the process of story comprehension, and how they are interrelated to adult's interaction behavior and the way they involve children in the story.

Therefore, based on the research desiderata reported in Miosga's study (2020), we took a media- *and* interaction-oriented approach in this study to explore how adult caregivers use the technical features of a picture-book app to involve children in the story. In doing so, we have deliberately concentrated on a commercial app since research still lacks of studies assessing the impact of commercial picture-book apps and their application in interaction on children's early literacy development. Previous studies often used specially designed apps and electronic books (Takacs et al., 2014) or they focused on app analyses across different countries, age groups, specific educational areas (Sari et al., 2019) or according to technical features and game activities (Sargeant, 2015; Serafini et al., 2016).

We first performed a media analysis of a picture-book app in order to identify the narrative potential of the medium. We based our analysis on the ViSAR model for picture-book app analysis, which we have applied in previous works (Müller-Brauers et al., 2020; Miosga et al., in press) and which we present in the methods section. The ViSAR model not only provides a theoretical basis to determine and compare app qualities in terms of narrative learning. Applying it to the narrative potential of apps and their interactive use in shared reading situations might also provide a promising approach in order to contribute new findings to the state of research and to resolve contradictions in current research findings. Hence, secondly, we assessed how far caregivers used the narrative potential of the app interactively during reading by investigating how often and in which mode adult readers referred to narrative animations verbally.

Hence, our analyses focused on the following research questions (RQs):

RQ 1. What kind of animations does the picture-book app provide? And how many animations can be identified as narrative animations?

RQ 2. How often (a) and in which content mode (b) do adult readers refer verbally to narrative animations in digital shared reading and do they exploit the narrative potential of the app?

To generate new research questions in the field of digital reading, we also conducted an explorative analysis focusing on the role of narrative animations and their interactive processing by the adult reader in children's early story comprehension by including exemplary data from children's story comprehension scores obtained after reading. We assumed that a high number of narrative animations linked to the story line would engage the child in the narrative more probably and therefore foster story comprehension (Korat, 2010; Sargeant, 2013, p. 32; Kao et al., 2016) because narrative skills benefit from joint engagement (Miosga, 2020) and thematic involvement (Pesco and Gagné, 2017; Grolig et al., 2019). Findings can provide a starting point for future research to assess what role the adult readers' verbal references play to specific app animations in children's early story comprehension.

## Methods

### Materials

The first research question in the study addressed the picture-book app “7 grummelige Grömmels und ein kleines Schwein” (“7 grumpy Grömmels and a little pig,” abbreviated to Grömmels below) by Wewer (2012). The app addresses children aged 3–4 years and was honored with a reward by the Stiftung Lesen and Leipziger Buchmesse (2013). As stated above, we intentionally selected a commercial app because previous studies often used specially designed apps and electronic books (Takacs et al., 2014). The app contains a reading function, sound effects (that can be activated or deactivated), a recording function, a coloring picture, and numerous hotspots in the form of green icons that constantly flash according to a programmed interval. Navigating animations for turning pages consist of small arrow symbols. The story, which is also available in book format, is about family and friendship. On a dark night, a pig arrives at a house where terrible monsters live—the hairy and grumpy Grömmels. The next morning, the seven Grömmels are not pleased when they notice their cheeky guest and threaten to eat it. By being kind and curious, however, the pig can save the day repeatedly and bring out the best in the Grömmels, so that they no longer wish to eat it.

### App Analysis (RQ 1)

**Figure 1:**
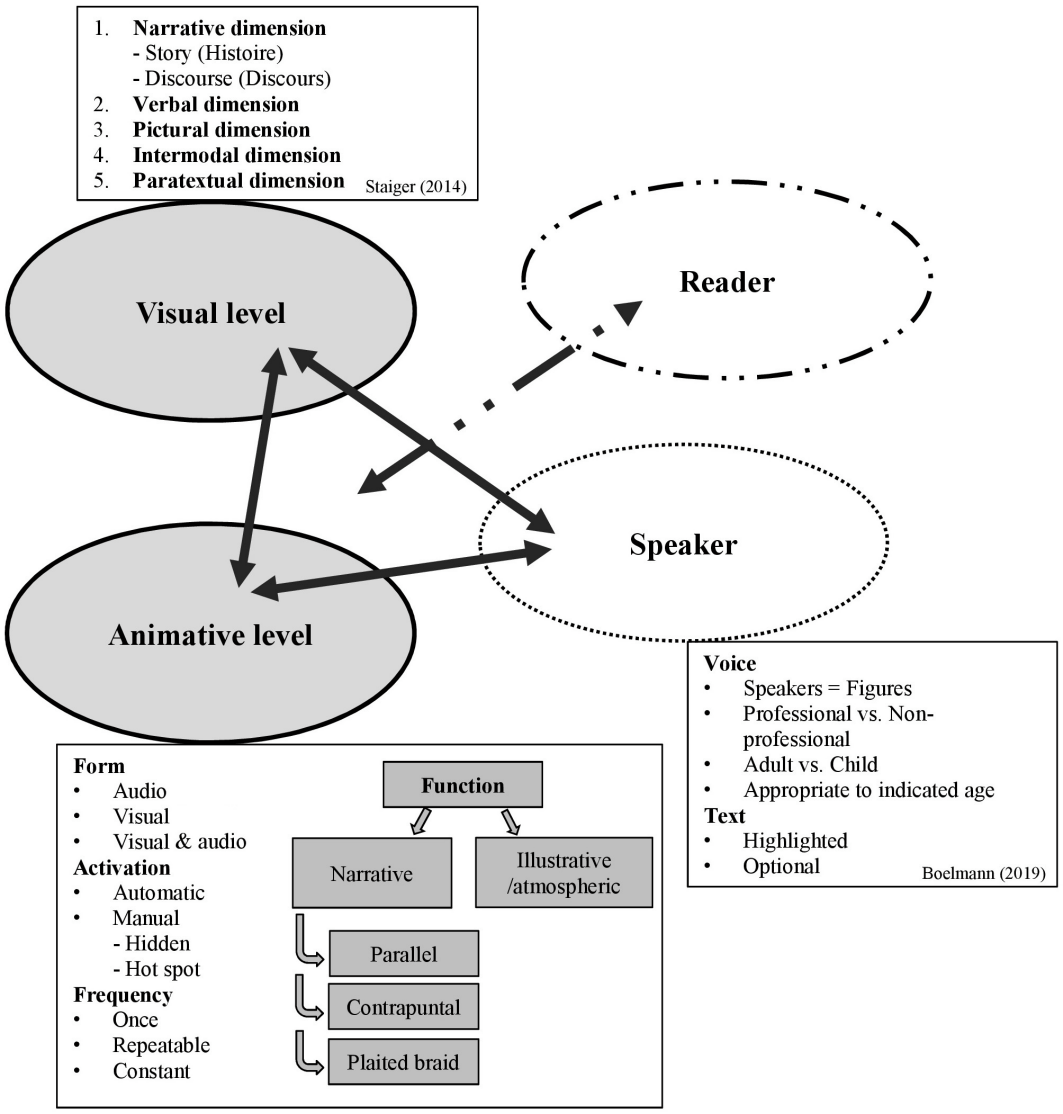
The ViSAR model for picture-book app analysis (Müller-Brauers et al., 2020, p. 174).

To address RQ 1, the app's animations were coded according to parameters suggested in the ViSAR model (Müller-Brauers et al., 2020). To obtain valid results, coding was controlled independently by a team of three researchers (for the coding scheme, see Supplementary Material). Furthermore, ambiguous cases were discussed in a team of four researchers. The ViSAR model, shown in Figure 1, integrates four interconnected levels of picture-book app analysis: visual, animative, reader, and speaker.

For validation purposes, we applied the model in previous work to different apps (Müller-Brauers et al., 2020; Miosga et al., in press) and the model was also tested in student's master's theses (e.g., von Wingerden, 2020) and presented and discussed on different conferences (Miosga and Müller-Brauers, 2019, 2020). In the course of the validation process, we shaped the difference between audio animations and the read aloud function by integrating the reader level. We also inserted the reader level to stress the impact of interaction in digital shared reading situations. To distinguish the model from exclusively media analysis, we also developed the ViSA-model, which does not contain the reader level (see Müller-Brauers et al., 2020).

The *visual level* of the ViSAR-model refers to Staiger's (2014, pp. 14–21) approach to a multimodal understanding of picture book analyses that integrates different analytical dimensions. The narrative dimension, for example, examines the function of the content and structure of the narrative: what is told in the story (plot, theme, space, figures, narrated time, etc.; see Kurwinkel, 2017) and how the story structure is unfolded (narrative perspective, figure speech, tense, and time sequences). The intermodal dimension highlights the interplay and interconnectedness of the verbal (text) and visual (images) code of picture books (Thiele, 2000). In the verbal dimension, analysis concentrates on the language input provided by the text in terms of text coherence, wording, syntax, linguistic style, and so forth^2^.

The second level, the *animative level*, focuses on analyzing the animations included in the app. Parameters include the form of animation (audio, visual, visual + audio), its activation, and the frequency in which the animation occurs:

- *Form:* visual (e.g., movements), audio (e.g., noises), visual + audio
- *Activation:* automatic (e.g., background music) or manual (on request after being clicked on) with manual animations being either hidden or controlled by hotspots
- *Frequency:* once, repeatable, or constant

The animative level also highlights the function that different animations can serve in terms of their potential for story comprehension. In reference to text–image relations (Thiele, 2000; Nikolajeva and Scott, 2006), the model distinguishes between three different subcategories:

1. *Parallel* (identical correlation of text and animated images/sounds). For example, the text describes a situation in which children are playing outside. Symmetrically, the animation displays identical actions or sounds when being activated.
2. *Contrapuntal* (inconsistent relation between text and animated images/sounds). For example, the text refers to a given scene in which children are playing outside until sunset. Is the sunset animated once, the animation is classified as parallel. If the animated sun rises again when reactivated, the animation is categorized as contrapuntal because this animation breaks the timeline and leads to a contradictory relation to the text (Müller-Brauers et al., 2020).
3. *Plaited braid* (complementary relation between text and animated images/sounds). Here, text and animated images/sounds present different but complementary information. Accordingly, one part of the relevant information is provided by the text, the other part by the visual or audio animation generating a meaningful context. For example, the text provides the information that the protagonist of the story had a lot of fun on that day, while the scene and the animations present the protagonist swimming, dancing, singing, and eating cake.

Animations can also be *illustrative* and thereby have an exclusively illustrative/atmospheric function (animating space visually: e.g., flying insects in trees; or evoking emotions auditorily: e.g., birds twittering in the background) and not be attached to the narrative dimension. In addition to illustrative and narrative animations, picture-book apps can also contain *navigating* animations that serve operative functions. Because these are not content-related, they are not addressed explicitly in the ViSAR model. However, by adding navigation applications, three types of animations can be accordingly distinguished: narrative, illustrative, and navigating animations.

The third level, the *reader level*, stresses the role of interaction in digital reading by pointing to the verbal and non-verbal behavior of the adult, caregiver, or any reading person in integrating the visual and animative level in the reading process. Our analysis focuses particularly on how the reader makes use of narrative animations to engage the child in the story, thereby possibly promoting the child's story comprehension^3^. The detailed coding scheme can be found in the Supplementary Material.

### Interaction Analysis (RQ 2)

The aim of the interaction analysis was twofold: When analyzing adult–child dyads (*n* = 12), we first aimed to determine how far the caregiver made use of the narrative potential of the app by referring to narrative animations verbally during reading. Therefore, we first assessed how often the caregiver referred to the different types of animations or hotspots (i.e., narrative or illustrative animations or hotspots), and how often she talked with the child about navigating the app^4^.

Secondly, we focused on the mode of verbal references. This means, we assessed on which content level the adult reader referred verbally to an animation. We included the mode of verbal references because the adult reader can elaborate in a narrative manner not only on narrative animations but also on illustrative and navigating animations. Inversely, adults can comment on all animation types in an operative mode, thus focusing on handling aspects of the animations. The codes *narrative* and *illustrative* were derived theoretically from the ViSAR model (Müller-Brauers et al., 2020), *navigating* from empirical evidence (e.g. Sargeant, 2015; Serafini et al., 2016; Miosga, 2020).

#### Participants

We analyzed data from a subsample of Miosga's (2020) participants containing 10 adults (5 mothers, 2 fathers, and 3 educational professionals) and 12 children (mean age: 36.33 months, range: 24–43 months, *SD* = 6.6 months). Participants were recruited via a questionnaire on digital media use in home environments and early childhood education in the greater Hanover area of North Germany. Parental consent was obtained for all participants^5^. Participants were monolingual German speakers with normal hearing and language abilities. Adult participants reported being familiar with digital media use and experienced in traditional as well as digital shared reading. Twelve adult–child dyads read the Grömmels app that provides a wide range of visual and audio animations. Interactions were videotaped for transcription and coding.

#### Coding

The adults' utterances were coded with the annotation tool ELAN (release 5.5, Max-Planck-Institute of Nijmegen). An utterance was defined as each phrase uttered by the adult or the child, not including reading the text (Miosga, 2020).

#### Quantity of Adults' Verbal References to Animations (RQ 2a)

Based on the ViSAR model, we coded adults' utterances according to verbal references to functional animation types (*narrative* vs. *illustrative*) and *navigating* animations (e.g., arrow icons to turn pages) provided by the picture-book app.

The utterances to narrative and illustrative animations were coded for each individual page of the app. The coding scheme included exactly one category for all narrative and one category for all illustrative animations for each page, respectively, even if a page presented more than one animation of each type. We did not further distinguish the different narrative animations (or illustrative animations, respectively) on each individual page. Thus, we captured narrative animations on 22 pages and illustrative animations on 13 pages because every page presented narrative animations, but only 13 pages also presented illustrative animations. Unclear or general references (e.g., “you can always click the green buttons when you see them”) as well as references to buttons for turning pages (navigating animations) were assessed in total (i.e., not distinguished between pages).

#### Modes of Verbal References to Animations (RQ 2b)

In this analysis, the mode of adults' verbal references defined as the mode of reference (*narrative, illustrative, operative*) to the different animations (*narrative* vs. *illustrative* vs. *navigating*) was identified within an analytical process. *Operative* refers in this analysis to the navigating functions of the app in the adults' comments. This variable assesses, thus, communicative references to navigating functions of the app. In an iterative process we observed that mixed categories also occurred, i.e., that adults referred to, for example, a narrative animation, but the reference was about operating aspects. For reasons of accuracy, we differentiated these categories in the coding process in, for example, narrative and operative-narrative categories. In this way, the following six categories were identified:

1. *Narrative:* The adult referred to a narrative animation narratively (e.g., “the pig is reading a book, isn't it?”)
2. *Illustrative:* The adult referred to an illustrative animation illustratively (e.g., “look, what is crawling on the wall?”)
3. *Operative-narrative:* The adult referred mainly operatively to a narrative animation, that means, for instance, including a story character (e.g., “can you tickle the pig”)
4. *Operative-illustrative:* The adult referred to an illustrative animation mainly operatively including an illustrative element (e.g., “will the dog reappear when you touch the sausage here?”)
5. *Narrative-navigating:* The adult referred narratively to an animation that serves navigating purposes (e.g., “shall we look at how to go on with the story?” when activating the button for turning pages)
6. *Operative:* The adult referred entirely operatively to an animation regardless of its function (narrative, illustrative, navigating) (e.g., “press the green button”)

The detailed coding scheme can be found in the Supplementary Material.

#### Story Comprehension

We also used data from the subsample of Miosga's study (2020) to assess children's story comprehension. The study had used a within-subject design to compare children's story comprehension under different media conditions. The data has been transcribed, anonymized, and processed in accordance with data protection guidelines. All participating researchers are obliged to comply with these guidelines. Adult–child dyads were randomly assigned to read either the traditional (analog) or app version (digital) first. Children's story comprehension was investigated once after reading the app or the book with a semistructured conversation stimulus (see also Parish-Morris et al., 2013; Reich et al., 2019). In story comprehension assessment, no global test is available with a full item analysis so that each assessment has to be designed according to the specific story. We realized this communicative approach by adjusting the stimulus and the questions to the content of the digital app used and to the age of the children. Doing so, children completed the story comprehension assessment after reading the app with the caregiver. In our study, we analyzed data from the digital media condition (*n* = 6).

Adults used questions to assess children's factual information extraction during reading by highlighting the main protagonists of the story or the motives for action: *Who is taking part in the story? Who lives in the house? What is the pig doing in the Grömmels' house? Why does the Grömmel want to eat the pig? Why is the pig allowed to stay with the Grömmels? Who are the Grömmels afraid of?* Children's answers were noted, recorded, and analyzed in terms of the proportion of correctly answered, incorrectly answered, and unanswered questions. Examples of correct answers given by the children are: “Who lives in the house?” *The Grömmels, the pig and the dog, Mommy Grömmel and children Grömmel, Dad, Mom, Baby and the pig*. Examples of incorrect answers given by the children are: “Why does the Grömmel want to eat the pig?” *Because he loves him very much, because he likes the piggy*. The coding scheme with further coding examples and descriptive statistics of the story comprehension categories is detailed in the Supplementary Material.

## Results

### App Analysis

The Grömmels picture-book app contains a total of 87 animations: 64 % are visual + audio; 18 %, exclusively visual; and 17 %, exclusively audio (all percentages rounded off). This means that 82 % of the total number of animations integrate sounds; 55 % are repeatable; 31 % run constantly when the noise function is switched on; and 14 % can be activated only once. In terms of activation, the majority of 64 % of animations are hotspots; 33 % start automatically; and only 2 % are hidden manually. Hotspots consist of a visual pointer projected in the form of a green icon that automatically reappears according to a technically predefined interval. We found that 79 % of the hotspots are visual + audio; 18 %, audio; and 3 %, visual. Hence, 96 % integrate sounds. About two thirds (61 %) of the hotspots have a narrative function; and more than one third (39 %), an illustrative and atmospheric function. This is equivalent to findings on the function of the animations: 62 % are narrative and 38 % illustrative. The group of narrative animations contains predominantly animations that function as a “parallelism” to the text (59 %). That means, they highlight identical information such as “One night a little pig walked into a house” (animation: door opens and a little pig enters) or “It switched the light on” (animation: the light turns on). Only 2 % of the narrative animations evoke a complementary relation to the text (plaited braid)—for example, “The Grömmel was very scared” (animation: the Grömmel family stand wide-eyed in the semidarkness of the background and tremble with fear). No animations in this picture-book app were coded as contrapuntal. A large number of animations (38 %) have illustrative and atmospheric functions (such as a dog wagging its tail or a fly circling around while making a loud buzzing noise).

#### Discussion of App Analysis

As a first interpretative step and reconsidering the theoretical background, we therefore conclude that the Grömmels picture-book app offers a high potential for early story comprehension because it includes a large number of various forms of narrative animations: 61 % of the animations have a narrative function; and at 59 %, almost all function as a parallelism. These animations have a high potential to support early story comprehension. In contrast, 39 % of the animations carry illustrative and atmospheric functions. These animations can distract from the storyline and may even be more likely to inhibit story comprehension. Similarly, the hotspots offer ambiguous potential: The app analysis shows that around two thirds of the animations are hotspots that appear temporarily, sequentially, and automatically. On the one hand, hotspots can support story comprehension if they appear at the right time and correspond to the narrative progress. On the other hand, they can distract from the storyline if they appear in an irregular manner. Additionally, the high number of animations that play with an auditory component (82 %) and have an illustrative function that mostly does not accompany the storyline can potentially distract from that storyline (Müller-Brauers et al., 2020; see also Parish-Morris et al., 2013; Smeets and Bus, 2013; Takacs et al., 2014; Yokota and Teale, 2014; Knopf, 2018).

However, there are also limits to our app analysis: The results of the app analysis based on the ViSAR model were verified by an independent researcher within the scope of an inter-reliability test who inspected 25 % of the animations. The level of agreement was 79 %. Maximum consensus was found in the frequency and activation categories. Most deviations were in the function category for the subcategories plaited braid and illustrative. The overall agreement on hotspots was slightly higher at 88 %. The frequency and total number categories showed the greatest consistency, whereas most deviations were found among functions—once more in the subcategories plaited braid and illustrative.

Ambiguous cases occurred especially for narrative parallel, plaited braid, and illustrative animations. Some animations could be interpreted as both plaited braid in the sense of referring to the Grömmels living in the house and illustrative in the sense of an atmospheric background supporting the scene. We finally classified these animations as illustrative because they tend to distract the reader from the narrative strand. Note, however, that counting animations on the basis of categories from the picture book analysis remains an interpretative process (Thiele, 2000).

In sum, we see that the app provides potential in terms of merits, but some demerits were demonstrated. The actual realization of this potential, however, needs to be illuminated by the following step.

### Interaction Analysis

Our second research question focused on (a) how far caregivers make use of the different animations in the app and (b) the mode of caregivers' verbal references to these animations. Our aim was to examine how adult readers exploit the narrative potential of the app. We therefore assessed how often caregivers addressed the different types of animations (*narrative, illustrative* and *navigating*) and in which ways they addressed them with regards to content. The numbers of utterances in each category were classified according to the coding schemes.

#### a) Quantity of Adults' Verbal References to Animations

This analysis initially determined the number of adult utterances on all narrative and illustrative animations per individual page, and then summed these up to a whole. Results showed that there were about twice as many utterances to narrative animations as to illustrative animations (see Tables 1, 2). The percentage ratio of solely narrative and illustrative animations without the other categories was about 70 % narrative to 29 % illustrative (see Table 1).

**Table 1:** Adult utterances to animation types (*n* = 12).

**Category**	**No. of utterances**			**No. of utterances/min**		
	***Md***	**Min**	**Max**	***Md***	**Min**	**Max**
Cumulative narrative	45.00	7	75	3.12	0.62	6.21
Cumulative illustrative	18.00	3	35	1.45	0.34	3.56
Cumulative narrative and illustrative	55.00	14	104	4.39	1.24	9.77
Navigating	12.00	0	35	0.87	0.00	2.87
Unclear/general	1.00	0	8	0.10	0.00	0.63
Total (narrative, illustrative, navigating, unclear/general)	74.00	22	122	5.26	2.51	12.31
Reading duration (min.)	12.54	8.75	17.68			

**Table 2:** Adult utterances (raw values) to animation types per page (*n* = 12).

**Page no./Type of animation**	***Md***	**Min**	**Max**
1 Illustrative	0.00	0	6
Narrative	0.50	0	5
2 Narrative	2.50	0	7
3 Narrative	2.50	0	8
4 Illustrative	3.50	0	10
Narrative	0.00	0	2
5 Narrative	3.00	0	6
6 Illustrative	2.00	0	10
Narrative	1.00	0	5
7 Illustrative	3.00	0	17
Narrative	1.00	0	5
8 Illustrative	1.00	0	8
Narrative	2.00	0	15
9 Illustrative	0.00	0	6
Narrative	1.50	0	8
10 Narrative	2.50	0	7
11 Narrative	0.50	0	3
12 Narrative	4.00	0	12
13 Illustrative	0.00	0	0
Narrative	1.50	0	8
14 Narrative	0.00	0	3
15 Narrative	2.00	0	5
16 Illustrative	0.00	0	3
Narrative	1.50	0	6
17 Illustrative	0.00	0	2
Narrative	1.00	0	4
18 Illustrative	0.00	0	1
Narrative	2.00	0	7
19 Illustrative	0.00	0	4
Narrative	0.00	0	3
20 Narrative	0.50	0	5
21 Illustrative	0.00	0	1
Narrative	1.50	0	4
22 Illustrative	0.00	0	2
Narrative	0.00	0	10

Expressed as a percentage of all utterances within the digital reading situation (narrative, illustrative, navigating, unclear/general), on average, about 55 % of adults' utterances were to narrative animations and 23 % to illustrative animations (19 % to navigating animations and 3 % unclear/general references).

Moreover, caregivers showed high variability in utterances to the different animation types (narrative: 19 to 82 %, illustrative: 8 to 42 %, navigating: 0 to 59 %, unclear/general: 0 to 12 %).

Table 1 presents the descriptive statistics. Because the duration of the digital reading situations differed between dyads, we also determined a ratio of each category in relation to total reading duration (utterances per min). Due to the small sample size and high variability, we report median and range as measures of central tendency.

The difference between the number of utterances to narrative or illustrative animations was also apparent *per page*. On some pages, adults related verbally to the animation types four times (a narrative animation on page 12), whereas on page 13, adults did not refer to any illustrative animation at all (see Table 2).

Some animations were elaborated more interactively than others: Most frequently, the illustrative animations on pages 4 and 7 were referred to as well as the narrative animations on pages 5 and 12 (all *Md* > 3.00).

Further analysis revealed that at the beginning of the digital reading situation (pages 1 to 11), the number of utterances to the app's narrative and illustrative animations was higher (*Md* = 35.00, range 9.0 to 84.0) than at the end of the story (pages 12 to 22, *Md* = 18.00, range 2.0 to 37.0). The amount of verbal references to narrative (*Md* = 16.5, range 5.0 to 49.0) and illustrative (*Md* = 17.0, range 2.0 to 35.0) animations on the first eleven pages of the app was comparable. On the following eleven pages, utterances to narrative animations stayed at an equivalent level (*Md* = 18, range 2.0 to 37.0), whereas the quantity of utterances to illustrative animations declined strongly (*Md* = 0.0, range 0.0 to 7.0).

#### Discussion of the Quantity of Adults' References

The analysis of the quantity of adults' verbal references to animations revealed that adult readers referred to narrative as well as to illustrative animations in a digital shared reading situation, and the number of utterances to narrative animations was about twice as high as those to illustrative animations. The number of references to navigating animations, however, was also substantial, nearly reaching the percentage of utterances to illustrative animations.

Further, when examining the adults' references to the animations on individual pages, some animations seemed to be especially attracting, and narrative animations might have an advantage here as well. The overall most frequently commented on animation was a narrative one (sleeping scene on page 12), whereas the one animation that was never commented by any dyad was an illustrative one (page 13, a fly flying through the room).

Considering the course of the reading situation, it becomes clear that references to illustrative animations strongly decreased during reading, whereas narrative animations were referred to quite consistently throughout the reading process, resulting in an overall lower amount of utterances to animations in general in the second part of the story. This result might be impacted by the reading process itself, and suggests that at the beginning of the reading situation, adults and children are testing the new functions of the different narrative or illustrative hotspot buttons. The operating modes might be exploited in this way, and the dyads focus on the functions and supplementary features that are not provided by a book, because they are somehow “new” and more interesting at the beginning.

Additionally, caregivers might arrange the entry to the story more extensively than the later progress of the reading situation—perhaps because they are aware that their child's concentration starts to fade or because they want to end the reading session. Especially the illustrative animations might be left out at the end of the story to save time in favor of narrative animations and a desire to concentrate on completing the story's plot. Further or alternatively, the illustrative animations might be less noteworthy or striking at the end of the story.

However, this result mirrors the fact that some dyads did not finish reading the app—indicating that interest decreased over the course of the reading situation. In this context, it is even more remarkable that a sleeping scene later in the app (page 12) was the most frequently referred to animation. It is possible that the content of this page had a major impact on this result because the scene might be linked closely to children's everyday lives at this age. App design might appreciate this content aspect.

Taken together, these findings suggest that narrative animations played a significant role in a digital reading situation with the Grömmels app because they were commented on more frequently than illustrative animations throughout the whole process of the reading situation.

Viewed together with the analysis of the app according to the ViSAR model, these results suggest that the full narrative potential presented by the app materiality (i.e., the number of narrative compared to illustrative animations) is fulfilled only partly in a digital reading situation. Indeed, on an interactive level, the percentage ratio of utterances to narrative and illustrative animations was about 70 to 30 %, therefore actually exceeding the ratio of the app materiality of 61 to 39 % from a narrative perspective. But, taking the reading situation as a whole and considering all utterances in the dyads' dialogues, talk about narrative animations was below what the app's materiality provides (i.e., about 55 %). Utterances on illustrative animations were even lower at only 23 %. The proportion of navigating and general references was similar (22 %). In other words, to some extent, adults referred to narrative animations to a slightly lesser extent than the app's overall narrative animations would suggest.

However, in this analysis, we did not focus on the way adults referred to the animations—that means, we did not examine the verbal content of these utterances. We concentrated only on the number of utterances to the different types of animation that adults referred to without considering their content. The content was examined in the following analysis.

#### b) Modes of Adults' Verbal References to Animations

Further analyses focused on the quality of verbal references by assessing the caregiver's mode of reference to the animations. We therefore determined the extent of narrative or illustrative elaborations on animations on the one hand and references to operative aspects of the app on the other hand in combination with the particular animation types based on the category system stated above (see Supplementary Material).

Descriptive statistics showed that the number of utterances in an operative mode of reference to navigating animations (operative) was highest compared to all other reference categories, and that this was apparent in raw values as well as in a ratio of each category to the individual reading duration of number of utterances per min. The range of utterances was high, indicating high variability in reading styles in the dyads. For descriptive statistics of raw values and a ratio of each category per duration of the reading situation, see Table 3.

**Table 3:** Mode of adult's references to animation types (*n* = 12).

**Category**	**No. of utterances**			**No. of utterances/min**		
	***Md***	**Min**	**Max**	***Md***	**Min**	**Max**
Narrative	25.50	2	45	2.15	0.18	4.48
Illustrative	6.00	0	28	0.45	0.00	2.03
Operative-narrative	5.50	0	16	0.42	0.00	1.17
Operative-illustrative	3.00	0	11	0.24	0.00	0.79
Narrative-navigating (turn page)	0.50	0	13	0.05	0.00	1.16
Operative	30.50	1	61	2.21	0.11	4.33
Utterances (total)	74.00	22	122	5.26	2.51	12.31
Reading duration (min.)	12.54	8.75	17.68			

Narrative utterances to narrative animations constituted 35 %, whereas illustrative utterances to illustrative animations represented 13 % of all utterances. Operative utterances to the animation types as a large category made up 37 %. Operative-narrative together with operative-illustrative and narrative-navigating constituted smaller categories at 7, 5, and 3 %, respectively (all values based on means and rounded off).

A Friedman test based on the ratio values indicated that across all categories, utterances differed significantly from each other, χ^2^(5) = 44.41, *p* < 0.001, *n* = 12. Pairwise comparisons with subsequent Wilcoxon tests revealed that operative did not differ significantly from narrative, but from all other categories (illustrative: *p* = 0.019, operative-narrative: *p* = 0.002, operative-illustrative: *p* = 0.003, narrative-navigating: *p* = 0.002). The category narrative also differed significantly from all other categories (all *p*s = 0.002). This revealed that the narrative mode of reference to narrative animations (narrative) was just as frequent as the operative category, but all other categories occurred significantly less often.

With regard to these categories, illustrative references to illustrative animations (illustrative) were used similarly frequently as operative references to narrative animations (operative-narrative, *p* = 0.11), but significantly more often than operative-illustrative and narrative-navigating (both *p*s < 0.05).

Adults used operative-narrative utterances significantly more often than operative-illustrative and narrative-navigating (both *p*s < 0.05). Operative references to illustrative animations (operative-illustrative) were less frequent, and the least frequent was referencing narrative in turning the pages (narrative-navigating). However, this comparison did not attain significance (*p* = 0.11).

Reliability was established by a second coder rating 25 % of the videos. Intercoder reliability was very good to perfect (Krippendorff's α ranged between *p* = 0.83 and *p* = 1.00 for all six categories).

#### Discussion of the Modes of Adults' Verbal References

Adults showed different modes of referencing during shared reading of the Grömmels app when considering the content of utterances. The most frequent were exclusively operative utterances to narrative, illustrative, and navigating animation types and, to the same amount, narrative references to narrative animations. Together with the results of the analysis of animation types and the app analysis, we can conclude that the narrative potential of the app was not exploited fully in a shared reading situation with a child. Even though adults referred to a large amount of narrative animations and often did so in a narrative mode, nearly the same amount of utterances addressed operating the animations.

Analysis 2b adds an important aspect regarding the content of the adults' references to the first two analyses (app materiality and the number of references to animation types): Although the app presents about 60 % narrative animations, and talk about narrative animations is slightly less frequent within a reading situation (55 % of all utterances), Analysis 2b reveals that narrative talk about narrative animations represents only 35 % of the dialogue. Illustrative talk about illustrative animations still constitutes 13 %, whereas the majority of utterances are about operating aspects (categories operative-narrative, operative-illustrative, and operative) representing 50 % of all utterances.

Thus, in digital reading situations, a large part of the talk addresses the digital nature of the app—talk that does not occur within an analog shared book reading situation. Illustrative talk to illustrative animations also takes place to the same extent as operative talk to narrative animations that also had some narrative aspects or referred to the story's characters. Operating aspects of illustrative animations and narrative comments on navigating were comparatively low.

#### Story Comprehension

In our explorative analysis focusing on children's story comprehension, we examined descriptively the children's answers (*correct, incorrect, no answer*) to the story comprehension questions in relation to the six coding categories of the mode of adults' utterances of the dyads and reading duration (*n* = 6).

The number of correctly answered questions was low overall (*correct answers: Md* = 1, range 0 to 5, *incorrect answers: Md* = 1, range 0 to 2, *no answer: Md* = 4, range 1 to 5). Therefore, we examined children's story comprehension and adults' verbal reference categories in a subsample of dyads in which adults used more narrative speech—thereby assuming that there would be a clearer relationship in these dyads. We performed a median split with the sample of the interaction analysis (*n* = 12) and identified this way six dyads with a higher ratio of the narrative category and analyzed the data of the dyads in which children had completed the story comprehension test (*Md* > 2.15, *n* = 4).

Table 4 reports the number of children's correctly answered, incorrectly answered, and unanswered questions for every single dyad as well as the respective reading duration and the mode of adults' utterances sorted by reading duration. For this analysis, and against the background that especially narrative references might foster the children's story comprehension, we summed up all categories other than narrative to one single category (“other than narrative”) for these four dyads.

**Table 4:** Results on children's story comprehension, reading duration and adults' utterances per dyad (*n* = 4).

**Dyad**	**Reading duration (min)**	**Utterances**	**Utterances/min**	**Narrative/min**	**Other than narrative/min**	**Correct answer**	**Incorrect answer**	**No answer**
1	9.83	121	12.31	0.36	7.83	1	0	5
2	11.16	85	7.62	0.29	5.38	1	1	4
3	12.70	69	5.43	0.41	3.23	5	1	1
4	17.68	122	6.90	0.37	4.36	0	2	4

A first analysis of the data suggested that children gave more incorrect answers on the comprehension of the story after longer digital reading situations as the number of incorrect responses was higher for those children experiencing a longer reading session (cf. Table 4).

With regard to the number of adults' overall utterances, the data shows that when the adults produced more utterances, the children's answers were less correct. This relationship is also mirrored in the number of no answers and the ratio of the quantity of all utterances per minute (utterances/min). The more utterances were produced in relation to the reading duration, the less children gave any answer.

The categories of the coding scheme suggest that the rate of narratives in relation to reading duration (narrative/min) might be related negatively to no answers, in the way that the more narrative utterances to narrative animations in the reading situation, the more children found an answer to the story comprehension questions (i.e., the lesser the number of “no answers”), although this observation is based on the behavior of a single dyad (no. 3). This child also showed the highest number of correct test responses. Conversely, a combined category of all five utterance types other than narrative might be related positively to no answers, in the way that the more utterances in the categories, the less children gave an answer. However, these descriptive results simply report data of four single app reading dyads, and have to be interpreted carefully as they sometimes rely on observations of a single dyad's behavior.

#### Discussion of the Story Comprehension

Taking story comprehension into account, descriptive results in a subsample of dyads in which the adults used a high rate of narrative utterances to narrative animations in relation to reading duration show that a higher number of utterances goes along with a lesser quantity of correct answers. Furthermore, when adults produced a higher number of utterances in relation to the duration of the reading situation, children more often left answers out.

All categories other than narrative related equally to children's left-out answers. In contrast, one dyad's child showed the best story comprehension after experiencing the most narrative utterances per minute in a reading session. Potentially, this observation indicates that narrative references of the adult lead to less left out answers and even more correct answers. However, this fact is in line with previous research that has shown narrative references to foster story comprehension (Korat, 2010; Sargeant, 2013, p. 32; Kao et al., 2016). This might possibly point out that our sample of four dyads also mirrors reading behavior that has been found in larger samples. Reading duration negatively affected the number of correctly answered questions in the way that the longer the reading duration, the higher the amount of incorrect answers in the children. Taken together, these results might indicate that story comprehension was lower when (a) reading duration was longer and (b) when adults made more utterances except for narrative utterances. However, these observations can offer only first suggestions regarding story comprehension in digital reading and point to the relevance of further research assessing the role of adult readers' verbal references to animations in processing digital stories.

## General Discussion and Practical Implications

In this study, we aimed to investigate how adult caregivers use animations of picture-book apps to involve children in stories from a media- as well as an interaction-oriented perspective. We first conducted an analysis of the app used in our study to determine the quantity of narrative animations (Analysis 1). Secondly, based on a quantitative interaction analysis, we examined how often (Analysis 2a) and in which content-related mode (Analysis 2b) adult readers make use of narrative animations to exploit the narrative potential of the app. Additionally, in an explorative analysis and as a starting point for further research, we also included children's story comprehension scores after digital reading to generate assumptions on the role of adults' verbal references for children's early story comprehension for a small subsample.

By adopting a two-fold approach, we aimed to close a research gap and combine an analysis of the medium with an analysis of interaction in dyads with the medium. Results suggest a promising number of narrative animations in the app, but adults do not fully exploit this potential in shared reading. Furthermore, derived from our explorative analysis on story comprehension, there are preliminary indicators that a prolonged reading duration might play a role in processing digital stories.

### Discussion of the Analyses

With regard to Analysis 1, note that from a theoretical point of view, the Grömmels app is promising with respect to children's story comprehension because it provides numerous narrative animations that can be used in shared reading to engage the child in the narrative. But, despite the high number of narrative animations, the app also contains technical features that may have a negative effect on story engagement, attention, and cognitive and verbal processing by providing a high proportion of automatically activated and constantly running visual + audio animations that cannot be deactivated (e.g., permanent buzzing flies or sounds of eating). Furthermore, adding a qualitative level of analysis, 32 % of the animations consist of multisectional sounds or visual images that take place at the same time or in quick succession and may lead to cognitive overload (e.g., the door opens with a squeaking noise and the little pig sticks its head through the door and grunts a couple of times). By the term “multisectional,” we refer to an animation consisting entirely of one or several subanimations that are interrelated regardless of whether they are linked by content, serve as complementary elements, or start simultaneously. Several visual + audio large-scale animations (9 %) operate all over the screen (e.g., the little pig sits on a ceiling lamp swinging from one side to the other) and several further animations (9 %) bear a risk of inducing auditory overload because they consist of multiple sound effects and background noises. Furthermore, the relatively high number of illustrative animations, hotspots, and animations played with an auditory component can potentially distract the reader.

Yet, a further descriptive level of analysis needs to be added to capture the app's functions in their entirety and to interpret its merits and demerits: the temporal appearance of the hotspots. During the reading process, it is notable that the hotspots appear arbitrarily in time and disorderly in terms of the story's progress. Therefore, the animations cannot be integrated into the narrative by the reader. Instead, a presentation of hotspots that corresponds to the story in terms of time and sequence would provide a beneficial context to the narrative.

With regard to Analyses 2 a and 2b, results from interaction analyses focusing on the type (Analysis 2a) and mode (Analysis 2b) of adults' verbal references show that adults refer most frequently to narrative animations, but not in a narrative way. This means that they do not use these animations primarily to engage the child in the story. Instead, they concentrate more on operating the animation. This also counts for illustrative and navigating animations, resulting in a predominance of operative references as the largest category with 49 %. A first analysis step (Analysis 2a) shows that adults refer twice as much to narrative as to illustrative animations, and percentages almost represent the ratio of the existing narrative to illustrative animations. But the content analysis in the second step (Analysis 2b) finally reveals that operating the app's animations is the most prominent mode of reference.

With regard to the story comprehension analysis, results provide first indicators that narrative references to narrative animations not necessarily guarantee that children are involved in the story, even though the small sample size restricts the generalization of these results to the population and limits interpreting the results in depth and reliability. We hope these results encourage future research for investigating this relationship thoroughly. There are also further limitations, such as we did not assess children's initial skill levels and include these in the analyses, although they have been shown to be relevant for story comprehension (Reese and Cox, 1999).

Moreover, the story comprehension assessment also requires productive language skills because children have to answer the questions. Thus, it may be considered to be quite demanding at this age, particularly as the characters' internal plans are relevant for half of the story comprehension questions. As Miosga (2020) has already pointed out, answers on factual information were superior to those on inference information.

At the same time, adults' concentration on the operative mode results in a high degree of operative talk leading to an extended reading duration that may affect children's story comprehension. Reading duration may have negatively affected the number of answered questions, suggesting that long reading sessions and a lot of operative input might raise the processing load. This suggestion is supported by an observation concerning the reading length: In the study by Miosga (2020) that compared analog and digital reading situations based on this app, reading duration differed significantly between both conditions; the digital reading session being around one third longer than the analog session.

Thus, we assume that the high amount of operative utterances combined with extended reading duration may have a negative effect on children's story comprehension, though, interpreting the results against the background of a small sample size that substantially limits drawing inferences and generalizations. The negative effect might potentially derive from a cognitive overload not only drawing children's attention away from the story and the thematic dimension of the reading, but also providing a less cognitively activating and content-related language input compared to analog book reading (Bus et al., 2015; Miosga, 2020). The high degree of operative talk may also affect the impact of dialogic reading routines that have proved to be advantageous for children's emergent literacy (Whitehurst and Lonigan, 1998). However, these analyses are considered only to be preliminary. Further analysis has to clarify whether these findings are replicable.

### Practical Implications and Outlook

The results of this study address several practice-related aspects: The app analysis suggests that in terms of story comprehension, the quality of an app results not only from the presence of numerous narrative animations but also from restricting the number of large-scale and multisectional animations that can carry cognitively overloading input and using a small, but targeted number of hotspots that correspond to the story in terms of time and sequence. The ViSAR model provides a theoretical basis that can be applied not only for study purposes but also in app design. Especially for developers of commercial apps, it might be interesting to know that “less can be more,” and that the nature and temporal design of animations are of importance; or, in other words, that it is more about the “how” and “when” of the animations than about the quantity.

Building on previous practical insights into what constitutes a “good” app for emergent literacy skills, the ViSAR model can enrich the search for conducive features, and it complements existing quality criteria and recommendations (Smeets and Bus, 2013). In particular, visual animations coordinated with the text have proved useful for word learning (Takacs and Bus, 2016). Interactive functions such as hotspots are also superior to pure text reading for word learning (Smeets and Bus, 2013). But what applies to word learning does not necessarily apply to story comprehension. Takacs (2015, pp. 144–145) formulates guidelines for app developers, parents, and educators on digital children's books based on the empirical evidence available to date and refers particularly to the integration of interactive animations and their effects on story comprehension. Therefore, the use of animated illustrations and the inclusion of sound and musical elements to illustrate the story is recommended. However, animations should be congruent with the text in terms of both content and timing. Animations can serve as a means of drawing the child's attention to individual details of the illustrations. Our results confirm these guidelines and can contribute differentiated recommendations on which type of animations has narrative potential.

A high proportion of narrative animations can be seen as beneficial because they enable the reader to involve the child in the story when being used in a narrative mode. At the same time, a high number of illustrative animations, hotspots, and animations that are played with an auditory component can potentially distract the reader (see also Parish-Morris et al., 2013; Smeets and Bus, 2013; Yokota and Teale, 2014). Overall, we conclude that when designing and developing picture-book apps, the proportion of illustrative and auditory animations should be reduced, and they should be used only sparingly and in a well-dosed manner (see also Smeets and Bus, 2013). The proportion of narrative parallel animations, in contrast, should be increased. Such animations might facilitate story comprehension in younger children and children with (linguistic or perceptual) impairments (see also Smeets et al., 2014), whereas numerous unconnected animations such as hotspots that appear arbitrarily in time and without relation to the story's progress can distract them. The reader cannot integrate such animations into the narrative. Instead, hotspots that correspond to the story in terms of time and sequence would provide a beneficial context to the narrative.

Previous quality criteria for picture-book apps in terms of story comprehension have been developed on the basis of empirical studies using specially designed apps. There, positive effects of digital story book reading on story comprehension in preschool age are often based on a non-commercial electronic book story designed especially for study purposes and optimized in accordance with literacy learning principles (Korat, 2009, 2010). Our study complements these results by providing recommendations for widely used commercial apps that have not been optimized deliberately for literacy learning. With regard to promoting narrative skills such as storytelling and story comprehension, our detailed interaction analyses preliminarily suggest that a high number of narrative animations does not lead to their integration into shared reading in a narrative way. On the contrary, hotspots lead to a large amount of operative talk, and large-scale moving images may lead to cognitive overload and shut down. This may apply especially when commercial apps are used for reading because these are not programmed according to psychological, pedagogical, or developmental criteria. Provided that the animations are especially designed as “narrative” with respect to the story, adults may be better able to refer to them; but when using commercial apps, the animations may not be a suitable resource for adults' reading dialogues.

Most importantly, these findings have practical implications on how to use narrative animations in interaction effectively. Guidelines for parents, practitioners, and caregivers should include recommendations on how to use narrative animations or animations in the narrative mode. For example, adults should take care that they refer to narrative animations in terms of the story and that they comment on the content. Moreover, illustrative animations can provide an opportunity to adapt adult's utterances to the child's everyday life experiences as well; in this case also through joint engagement and content-related, decontextualized language input that fosters story comprehension. Adults support story comprehension when making sure to link dialogues on illustrative animation back to the story. In sum, quality criteria for apps should be differentiated according to the target perspective and the area of support. For this very reason, it is essential for the adult reference person to take a reflected approach to the animations.

Illustrations in picture storybooks are multifunctional and need to be interpreted (Schickedanz and Collins, 2012). This may also be applied to electronic features. Practitioners should consider the ways in which technology can help support children in specific areas. They should try out different interactive apps and experiment with the interactive features. Self-reflection on how these functions affect their reading of the text will lead to a more conscious approach. Electronic resources can thus deliver a potential benefit compared to analog reading. The present study contributes to research on the nature of these potentials.

However, our interaction studies are subject to some further limitations: First, the age range of the children was quite high, making our sample rather heterogeneous. Because parents attune to their children, their reading behavior is influenced by age (e.g., Barachetti and Lavelli, 2011; see also Strouse and Ganea, 2017). Moreover, children's story comprehension is better at an older age (e.g., Parish-Morris et al., 2013), and this might have impacted on our story comprehension results. The book and the app might also have been quite demanding and too long for some of the children in the age group tested here. Hence, it would be desirable to examine more homogeneous samples of slightly older children with this app.

Another limitation is inherent to the chosen method of quantitative content analysis because categories represent a selected choice and contain rather fixed boundaries compared to other methods. Moreover, we concentrated on examining talk referring to animations and hotspots in this study without further considering the talk outside these animations such as comments on the whole scenes or utterances about the story itself. Additionally, the content analysis distinguished only two broad categories: adults' narrative and operative utterances. It would be worth expanding these analyses and considering precisely the adults' verbal behavior in digital book reading. Miosga (2020) has already shown that the content of utterances differs between digital and analog book reading, and such analyses might be extended to other categories that have been identified as fostering language development such as decontextualized language.

Further research on the use of animations in interactive reading situations might not only consider both children and caregivers, but also include conversational analysis in order to display the mutual process of attunement and multimodal communication. As desiderata for literacy learning, we also recommend a further validation of the ViSAR model and the development of apps on the basis of the ViSAR model testing them systematically in the field. We strongly recommend examining both the digital medium and its application in interaction in order to further analyze the potential of digital picture-book apps and shared reading situations.

## Data Availability Statement

The datasets presented in this article are not readily available because the data used in this article are processed under the informed consent, which excludes passing the data on. Requests to further information on the datasets should be directed to Christiane Miosga, christiane.miosga@ifs.uni-hannover.de.

## Ethics Statement

Ethical review and approval was not required for the study on human participants in accordance with the local legislation and institutional requirements. Written informed consent to participate in this study was provided by the participants' legal guardian/next of kin.

## Author Contributions

CM-B, CM, and SF contributed to conception and design of the study. SF, CM-B, and AM performed the analyses and statistical analyses. SF, AM, IP, CM-B, and CM wrote the methods chapter of the manuscript and discussion of the manuscript. SF, AM, and IP wrote the results. All authors contributed to the section practical implication and outlook. All authors also contributed to manuscript revision, read, and approved the submitted version.

## Conflict of Interest

The authors declare that the research was conducted in the absence of any commercial or financial relationships that could be construed as a potential conflict of interest.

## Footnotes

^1^By “multimodality,” we mean the combination of phonetic with mimic, gestural and other body-related components, and thus auditory and visual ‘modalities’ in face-to-face interaction (Steinseifer, 2011, p. 165).

^2^The visual design, color, picture space, typography, and pictorial composition (Staiger, 2014) frame the pictorial dimension of the analysis. Finally, the paratextual and material dimension highlights the material constitution of a picture book. These are the book format, cover and endpapers, type of paper, and so forth.

^3^The fourth level of the ViSAR model encompasses the *speaker* included in an app as part of the read-aloud function (Boelmann, 2019, p. 257). The speaker transfers the written text and therefore the narrative into oral language. Thereby, the speaker adopts the role of a narrator or reader to the child interacting with the app. For our analysis, the speaker is irrelevant. Therefore, we do not pursue this aspect further.

^4^Animations activated by a hotspot are not discriminated from other animations in the interaction analysis. Hence, in the following reports on the interaction analysis, we refer only to the term animation.

^5^All subjects gave written informed consent in accordance with the Declaration of Helsinki.
