# Supplementary material for: Narrative Potential of Picture-Book Apps: A Media- and Interaction-Oriented Study
Source: Front Psychol. 2020 Dec 2;11:593482. doi: 10.3389/fpsyg.2020.593482 (PMC7738561; doi:10.3389/fpsyg.2020.593482)
Supplement: Supplementary file 1 [file Data_Sheet_1.pdf]

Supplementary Material

1 Coding scheme – app analysis based on ViSAR model (Müller-Brauers et al. in press)

Category	Explanation	Examples
**animations**	- activatable or activated visual images that either move or are movable<br>- activatable or activated sounds	- an owl falls from a perch after being clicked on<br>- after a click, the sun goes down and the light is dimmed in the scenery<br>- background noises of children playing<br>- a football is rotating in the air after being clicked on
**form**	- supercategory for the outer form of animations<br>- subcategories are audio, visual and visual+audio animations	
**audio**	background noises, sounds, music, noises and voices except the voice of the narrator	- background noises of children playing<br>- a rubber duck is squeaking<br>- owl calls
**visual**	movable or moving images in contrast to the static scenery of the picture-book app	- an owl is flying away<br>- a character kicks a football<br>- a character scratches its chin
**visual + audio**	combination of audio- and visual elements that belong together and appear simultaneously or in quick succession	- a character presses the doorhandle and makes a movement/ gesture with its arm<br>- a squeaking door opens<br>- a wobbling and creaking lamp
**activation**	- supercategory for the type of starting the animations<br>- subcategories are automatic, manual activatable visible animations (so called „hotspots“) and manual hidden animations	
**automatic**	animation starts automatically as soon as the respective „page“ opens and can possibly be turned off in the app menu (e. g. background noises) but cannot be stopped, interrupted or clicked on separately	- background noises of children playing<br>- birds‘ twittering in the background
**hotspots**	animation starts after being clicked on manually with the help of a visual cue or pointer	- a visual pointer appears on a character and after being clicked on, the character moves and performs an action
**manual hidden**	animation starts manually after being clicked on without the help of a visual cue or pointer and can only be found by trial and error	- a character moves or performs an action after being clicked on without the help of a visual pointer
**frequency**	- supercategory for the frequency of animations<br>- subcategories are once, repeatable and constant	
**once**	animation appears only once	- an animal performs an action one time only<br>- a bird flies away and this action is irreversible
**repeatable**	animation can be reactivated after it has finished its movement/sound	- a football rotates in the air repeatedly after being clicked on again and again
**constant**	animation runs automatically or constantly after being clicked on without an interruption until the „page“ is finished or the picture-book app is exited	- background noises of children playing<br>- birds‘ twittering in the background
**function**	- supercategory for the content-related function of animations<br>- subcategories are parallel, plaited braid, contrapuntal and illustrative	
**parallel**	animations appear with an identical correlation of text and animated images/ sounds	- text: “The child is playing with his friends.“, animation: background noises of children playing<br>- text: “The child grimaced in front of the mirror.“, animation: A character sticks out his tongue.
**plaited braid**	animations appear with a complementary relation between text and animated images/ sounds and	- text: “The uncle thought it over. ‘We could sit at the window and watch the moon.’ he proposed.”, animation: The child approaches

Category	Explanation	Examples
	complement each other to a meaningful statement comparable to a “plaited braid” (Thiele, 2000)	his uncle standing at the window with the moon outside in the sky.<br>- text: “At this point the child has already fallen asleep.”, animation: The child is cuddling in the arms of its uncle.<br>- text: “Findus had a lot of fun that day.”, animation: Findus is swimming, laughing, playing and eating cake.
**contrapuntal**	animations appear with an inconsistent relation between text and animated images/ sounds and evoke a contrast between text and images/ sounds that can either be humorous or irritating	- text: “The child played with his friend until the sun went down.”, animation: The sun goes down and the light is dimmed. After that, the sun can be clicked on again so that the sun rises again and the light goes on again.
**illustrative**	audio, visual or visual + audio animations that appear to shape the mood and atmosphere of the scenery	- birds‘ twittering in the background<br>- a rubber duck that is not part of the story is squeaking<br>- a bee is sitting on a flower and makes some „buzzing“ noise

Reference:

Müller-Brauers, C., Boelmann J. M., Miosga, C., and Potthast, I. (in press). “Digital children’s literature in the interplay of visuality and animation - A model to analyze picture book apps and their potential for children’s story comprehension,” in International perspectives on digital media and early literacy: The impact of digital devices on learning, language acquisition and social interaction, eds K. J. Rohlfing, and C. Müller-Brauers (London, England: Routledge).
