# Supplementary material for: Narrative Potential of Picture-Book Apps: A Media- and Interaction-Oriented Study
Source: Front Psychol. 2020 Dec 2;11:593482. doi: 10.3389/fpsyg.2020.593482 (PMC7738561; doi:10.3389/fpsyg.2020.593482)
Supplement: Supplementary file 2 [file Data_Sheet_2.pdf]

Supplementary Material

2 Coding scheme – adults' ways of reference to types of animations/hotspots

Category	Content of reference & type of animation/hotspot	Explanation	Examples
**Narrative**	Narrative (content) & narrative (type)	Narrative reference to an animation/hotspot that is classified as narrative (i.e., the animation/hotspot strengthens or completes the narration of the story line)	"What is the pig doing?"<br>"They are looking at what the pig is doing, aren't they?"<br>"Look who is coming" (the pig)<br>"There it is standing in front of the mirror, isn't it?" (the pig)<br>"Is reading a book, isn't it" (the pig)<br>"Is somebody laughing?" (the pig)<br>"They are scratching its back?" (the monster's back)<br>"Oh, they are all snoring" (bed scene)
**Illustrative**	Illustrative (content) & illustrative (type)	Illustrative reference to an animation/hotspot that is classified as illustrative (i.e., the animation/hotspot has exclusively an illustrative/atmospheric function and is not attached to the narrative dimension).	"It's hanging loose, isn't it" (key/comb)<br>"Oh, fell down" (comb)<br>"This is funny" (the slippers can be thrown on the screen)<br>"What is he doing?" (illustrative)<br>"What is crawling up the wall?" (ladybug)<br>"Oops what happens to the slippers"
**Operative-narrative**	Operative-narrative (content) & narrative (type)	*Predominantly* operative reference to a narrative animation/hotspot (i.e., only aspects of handling narrative animations/hotspots are referred to). The caregiver may refer to these aspects in terms of her own narrative that is not the same narrative of the story, or she takes up a character of the story.	"Press the pig"<br>"Can you tickle it, tickle the pig?"<br>"Switch on the light" (as the pig does)<br>"Do the nose of the pig"<br>"The big Grömmel has something to say, I think"
**Operative-illustrative**	Operative -narrative (content) & illustrative (type)	*Predominantly* operative reference to an illustrative animation/ hotspot (i.e., only aspects of handling illustrative animations/hotspots are referred to). The caregiver may refer to these aspects in terms of her own narrative that is not the same narrative of the story, or she takes up a character of the story.	"Can you do something with that" (illustrative animation/ hotspot)<br>"This is funny" (the function of an illustrative animation/ hotspot)<br>"Try (pushing) the sausage, maybe the dog will show up"<br>"Can you do the dog?"<br>"You want to put them there?" (the slippers)
**Narrative-navigating**	Narrative (content) & navigating (type)	Narrative reference to an operative hotspot (turning the page). The caregiver develops her own narrative relating to turning the page.	"Shall we look at the story how to go on?"<br>"Oh, we did not continue telling the story here" (turning two pages accidentally)<br>"So..." (while turning the page)
**Operative**	a) Operative (content) & navigating (type)	a) Operative reference to a navigating type of hotspot (turning the page).	a) "Let's continue"<br>"You're in a hurry"<br>"That was fast, wasn't it?" (all examples: turning the page)<br>"Can you turn the page, please?"<br>"I think, we have turned one page too far now"<br>"You keep going?"<br>"Now you have turned backwards"<br>"Then you turn forward again"
	b) Operative (content) & narrative or illustrative (type)	b) *Mere* operative reference to a narrative or illustrative animation/hotspot.<br>Only operative features are referred to, without taking up a character or developing a new narrative (cf. categories operative-narrative and operative-illustrative).	b)"Something's shining there, isn't it?"<br>"Touch it"<br>"Once again?"<br>"Try it"<br>"Is something happening there?"<br>"Look, there is something else to press"
