# Supplementary material for: Narrative Potential of Picture-Book Apps: A Media- and Interaction-Oriented Study
Source: Front Psychol. 2020 Dec 2;11:593482. doi: 10.3389/fpsyg.2020.593482 (PMC7738561; doi:10.3389/fpsyg.2020.593482)
Supplement: Supplementary file 3 [file Data_Sheet_3.pdf]

Supplementary Material

3 Story comprehension test

All questions/ items	Coding examples of correct and wrong answers		Statistic per item (n/% of correct and false responses)		
**Who is taking part in the story?**	examples of correct answers:<br>- the pig<br>- Baby Grömmel, Dad Grömmel, Mum Grömmel and the little pig	examples of wrong answers:<br>- the grandma<br>- my aunt Lillie	statistics for correct responses:<br>16,67 % (n=1)	statistics for false responses:<br>0 % (n=0)	statistics for no responses:<br>83,33 % (n=5)
**Who lives in the house?**	examples of correct answers:<br>- the Grömmels<br>- the pig, the dog	examples of wrong answers:<br>- the monster<br>- the Easter Bunny	statistics for correct responses:<br>16,67 % (n=1)	statistics for false responses:<br>50 % (n=3)	statistics for no responses:<br>83,33 % (n=5)
**What is the pig doing in the Grömmels' house?**	examples of correct answers:<br>- goes to the toilet<br>- has eaten lunch	examples of wrong answers:<br>- hits them	statistics for correct responses:<br>16,67 % (n=1)	statistics for false responses:<br>0 % (n=0)	statistics for no responses:<br>83,33 % (n=5)
**Why does the Grömmel want to eat the pig?**	examples of correct answers:<br>- Because he is angry.<br>- Because he fooled around all the time.	examples of wrong answers:<br>- Because he loves it so much.<br>- Because he loves the piggy.	statistics for correct responses:<br>16,67 % (n=1)	statistics for false responses:<br>0 % (n=0)	statistics for no responses:<br>83,33 % (n=5)
**Why is the pig allowed to stay with the Grömmels?**	examples of correct answers:<br>- Because it loves them so much.	examples of wrong answers:<br>- No. I want to go home, the pig says.	statistics for correct responses:<br>16,67 % (n=1)	statistics for false responses:<br>0 % (n=0)	statistics for no responses:<br>83,33 % (n=5)
**Who are the Grömmels afraid of?**	examples of correct answers:<br>- of the monster<br>- of the … so big (shows with its hand in the air)	examples of wrong answers:<br>- piggy	statistics for correct responses:<br>33,33 % (n=2)	statistics for false responses:<br>33,33 % (n=2)	statistics for no responses:<br>33,33 % (n=2)
